# Structural basis for ATG9A recruitment to the ULK1 complex in mitophagy initiation

**DOI:** 10.1126/sciadv.adg2997

**Published:** 2023-02-15

**Authors:** Xuefeng Ren, Thanh N. Nguyen, Wai Kit Lam, Cosmo Z. Buffalo, Michael Lazarou, Adam L. Yokom, James H. Hurley

**Affiliations:** ^1^ Department of Molecular and Cell Biology, University of California, Berkeley, Berkeley, CA 94720, USA.; ^2^ California Institute for Quantitative Biosciences, University of California, Berkeley, Berkeley, CA 94720, USA.; ^3^ Aligning Science Across Parkinson’s (ASAP) Collaborative Research Network, Chevy Chase, MD 20815, USA.; ^4^ Walter and Eliza Hall Institute of Medical Research, Melbourne, Australia.; ^5^ Department of Biochemistry and Molecular Biology, Biomedicine Discovery Institute, Monash University, Melbourne, Australia.; ^6^ Department of Medical Biology, University of Melbourne, Melbourne, Victoria, Australia.; ^7^ Helen Wills Neuroscience Institute, University of California, Berkeley, Berkeley, CA 94720, USA.

## Abstract

The assembly of the autophagy initiation machinery nucleates autophagosome biogenesis, including in the PINK1- and Parkin-dependent mitophagy pathway implicated in Parkinson’s disease. The structural interaction between the sole transmembrane autophagy protein, autophagy-related protein 9A (ATG9A), and components of the Unc-51–like autophagy activating kinase (ULK1) complex is one of the major missing links needed to complete a structural map of autophagy initiation. We determined the 2.4-Å x-ray crystallographic structure of the ternary structure of ATG9A carboxyl-terminal tail bound to the ATG13:ATG101 Hop1/Rev7/Mad2 (HORMA) dimer, which is part of the ULK1 complex. We term the interacting portion of the extreme carboxyl-terminal part of the ATG9A tail the “HORMA dimer–interacting region” (HDIR). This structure shows that the HDIR binds to the HORMA domain of ATG101 by β sheet complementation such that the ATG9A tail resides in a deep cleft at the ATG13:ATG101 interface. Disruption of this complex in cells impairs damage-induced PINK1/Parkin mitophagy mediated by the cargo receptor NDP52.

## Teaser

Trimeric structure reveals the crucial interaction between the ULK1 and ATG9 complex vital for NDP52 dependent mitophagy.

## INTRODUCTION

Macroautophagy (henceforth autophagy) is a conserved cellular degradative process that maintains cellular homeostasis under a variety of stress conditions including starvation, mitochondrial damage, and microbial invasion. One of the major functions of autophagy is to selectively target and degrade various unneeded or dangerous cellular cargoes. Dysfunction of selective autophagy and the consequent accumulation of problematic cargoes contribute to a multitude of human disease (*1*). Parkinson’s disease (PD) is one of the clearest examples of an autophagy defect linked to human disease. In *PRKN* and *PINK1* patients with PD, the failure to clear damaged mitochondria via mitophagy is thought to contribute to disease due to the progressive loss of dopaminergic neurons in the substantia nigra pars compacta (*2*, *3*). Selective autophagy proceeds from the recognition of cargoes, formation of autophagy initiation sites, de novo synthesis of a double lipid bilayer termed the phagophore (or isolation membrane), maturation of the phagophore into a closed autophagosome that sequesters cargo, and, lastly, autophagosome-lysosome fusion leading to cargo degradation (*4*–*6*). A set of core autophagy initiation proteins bridge cargo recognition to isolation membrane biogenesis and elongation into the cup-shaped phagophore (*4*, *5*). In mammalian autophagy, the Unc-51–like autophagy activating kinase (ULK1) complex, PI3KC3 complex I (PI3KC3-C1), ATG12-5-16L1 autophagy-related protein 8 (ATG8) conjugation machinery, ATG2A/B, and ATG9A are all fundamental to autophagosome formation (*7*). How these complexes interact to trigger initiation has been challenging to dissect due to the presence of overlapping and partially redundant activities and the large size and dynamic character of the protein complexes.

The ULK1 complex is composed of four proteins: the ULK1 kinase, ATG13, ATG101, and focal adhesion kinase family interacting protein of 200 kDa (FIP200) (*8*–*11*). The ULK1 and ULK2 kinases regulate much of the autophagy pathway through phosphorylation events (*12*). The nonkinase subunits of the ULK1 complex have numerous scaffolding and bridging roles, some of which act upstream of ULK kinase activity. FIP200 is recruited by autophagic cargo receptors to mark sites of autophagy initiation and scaffolds the assembly of the ULK1 complex (*13*–*16*). Cargo receptors including p62 and Nuclear domain 10 protein 52 (NDP52) engage with the C-terminal coiled coil and Claw domains of FIP200 (*13*–*15*). These interactions place the ULK1 complex in an upstream position in many forms of selective autophagy, such that it is thought to be responsible for bridging the rest of the autophagy initiation machinery (*17*).

**Fig. 1.**
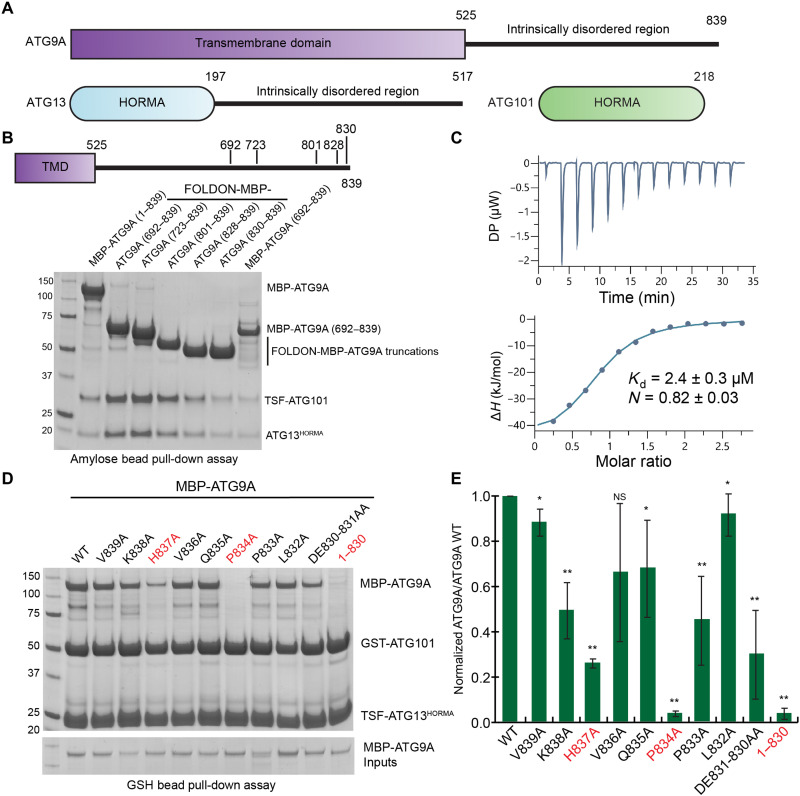
Domain arrangement and ATG9A C-terminal tail pull-downs. (**A**) Domain schematic of autophagy-related protein 9A (ATG9A), ATG13, and ATG101. HORMA, Hop1/Rev7/Mad2; TMD, transmembrane domain. (**B**) MBP pull-down assay using recombinant MBP-ATG9A in DDM/CHS micelles, MBP-FOLDON-ATG9A, and MBP-ATG9A constructs bound to amylose resin. Incubated with ATG13^HORMA^/TwinStrep-Flag (TSF)–ATG101 and visualized on 4 to 12% SDS–polyacrylamide gel electrophoresis (SDS-PAGE) gel. (**C**) Isothermal titration calorimetry (ITC) of MBP-ATG9A (801–839) and ATG13^HORMA^:TSF-ATG101. DP, differential power. (**D**) Glutathione *S*-transferase (GST) pull-down assay using purified TSF-ATG13^HORMA^:GST-ATG101 incubated with lysate containing ATG9A wild type (WT) or mutants. Bound protein was run on 4 to 12% SDS-PAGE gel. (**E**) Quantification of GST pull-down assays from (D) showing SD (*n* = 3). Two-sample *t* test was used to compare wild-type to mutant pull-down results. *P* values are as follows: *P < 0.1; **P < 0.05; NS, not significant.

ATG13 translocation to initiation sites is a key early step in both starvation-induced autophagy and mitophagy (*18*, *19*). The ATG13 and ATG101 subunits of the ULK1 complex form a heteromeric dimer through homologous Hop1/Rev7/Mad2 (HORMA) domains (Fig. 1A) (*20*, *21*). HORMA domains contain a “safety belt,” which conformationally regulates binding to protein proteins (*22*). ATG101 contains a protruding Trp-Phe (WF) finger motif, which is important for autophagy initiation (*20*, *21*, *23*), but whose precise function is unknown. In addition to a HORMA domain, ATG13 contains a C-terminal intrinsically disordered region (IDR) of ~300 residues. This IDR is responsible for interaction with FIP200 and the ULK1 kinase (*24*–*26*).

ATG9A is the only known ubiquitously expressed transmembrane protein of the autophagy initiation cascade in mammals (*27*). ATG9A forms Golgi-derived vesicles, which are recruited to autophagy initiation sites through a complex trafficking process (*28*–*32*). Global knockout (KO) of *ATG9A* in mice results in impaired autophagosome biogenesis and accumulation of p62 aggregates (*33*), and conditional KO in the brain leads to progressive neurodegeneration (*34*). Structurally, ATG9A is composed of two distinct domains, a transmembrane domain (TMD; 1–525) and a C-terminal IDR (526–839) (Fig. 1A). The TMD of ATG9A forms an interlocked trimer and functions as a lipid scramblase (*35*–*37*). ATG9A distributes incoming endoplasmic reticulum (ER)–synthesized lipids, transported via ATG2 proteins, across the growing phagophore from the outer to the inner leaflet (*38*, *39*). The IDR region of ATG9A contains sites of regulatory signaling including phosphorylation via TANK-binding kinase 1 (TBK1) and ULK1, ubiquitination, and direct protein-protein interactions (*40*–*42*). It has been shown that yeast Atg9 is capable of acting as a seed for phagophore initiation (*43*), and it seems reasonable to expect that mammalian ATG9A might do the same. To carry out any of their lipid transfer, regulatory, assembly, or putative seeding functions, ATG9A vesicles must first be recruited to sites of autophagy initiation, the focus of the present study.

ATG9A, ATG13, and ATG101 form a multifunctional hub at an early stage of starvation-induced autophagy and mitophagy initiation (*18*, *19*, *44*). A Biotinylation identification (BioID) mass spectroscopy approach revealed that ATG13:ATG101 can recruit ATG9A during p62-dependent autophagy, independent of FIP200 and ULK1 (*45*). Deletion of ATG13 or ATG101 led to a mislocalization of ATG9A, leading, in turn, to an accumulation of p62 aggregates identical to the ATG9A KO phenotype (*33*, *45*). This study highlighted the importance of the ATG9A, ATG13, and ATG101 nexus and focused our attention on this subnode of the autophagy interaction network. Clearance of damaged mitochondria requires both the ULK1 complex and ATG9A to promote efficient mitophagy (*46*). During PINK1/Parkin-dependent mitophagy, the ubiquitination of outer mitochondrial membrane proteins promotes recruitment of Optineurin (OPTN), NDP52, and p62 (*47*–*49*). However, TBK1-phoshorylated OPTN has been reported to directly recruit ATG9A during mitophagy, thereby bridging cargo to ATG9A vesicles in a manner that could potentially bypass the need for ATG13 to recruit ATG9A (*50*, *51*). This contrasts with the strict dependence of NDP52-mediated selective autophagy on the ULK1 complex for initiation, which led us to focus on the role of the ATG9A:ATG13:ATG101 complex in NDP52-dependent PINK1/Parkin mitophagy. Here, we have identified the binding interface between ATG9A, ATG13, and ATG101. We term the region of the extreme C terminus of ATG9A that binds to the ATG13:ATG101 HORMA dimer the “HORMA dimer–interacting region” (HDIR). We determined the structure of the human ATG9A HDIR bound to the ATG13:ATG101 HORMA dimer and confirmed that the interface functions in NDP52-dependent mitophagy.

## RESULTS

### ATG9A tail interacts with the ATG13:ATG101 HORMA dimer

To investigate the interaction between ATG9A and the ULK1 complex, ATG9A, ATG13, and ATG101 proteins were overexpressed and purified via mammalian human embryonic kidney (HEK) 293GnTi cells. The HORMA domain (1–197) of ATG13 (henceforth called ATG13^HORMA^) was purified along with ATG101. An amylose bead pull-down assay using full-length and N-terminal truncations of ATG9A as bait to recruit ATG13^HORMA^:ATG101 was performed (Fig. 1B). Full-length Maltose-binding protein (MBP)-ATG9A in detergent micelles strongly bound ATG13^HORMA^:ATG101. To study the isolated soluble C-terminal domain of ATG9A as a trimeric assembly, we engineered a FOLDON domain N-terminal to the ATG9A C-terminal constructs. FOLDON is a small 8-kDa N-terminal domain of T4 fibritin, which intrinsically forms trimers (*52*). MBP-FOLDON-ATG9A C-terminal Doman (CTD) constructs containing 692–839, 723–839, 801–839, 828–839, and 830–839 all recruited ATG13^HORMA^:ATG101 (Fig. 1B). These data show that the last nine residues of ATG9A (termed the ATG9A tail) are sufficient to recruit ATG13^HORMA^:ATG101 (Fig. 1B). Monomeric MBP-ATG9A (692–839) also recruited the ATG13^HORMA^:ATG101; therefore, the trimeric assembly is not required. We designate human ATG9A residues 830–839 as the “HDIR”.

To determine the affinity of the ATG9A HDIR for ATG13^HORMA^:ATG101, we used isothermal titration calorimetry (ITC). Serial injection of MBP-ATG9A (801–839) into a cell containing ATG13^HORMA^:ATG101 resulted in a dissociation constant (*K*_d_) of 2.4 ± 0.3 μM (Fig. 1C). This assay showed equal stoichiometry between the ATG13^HORMA^:ATG101 dimer and ATG9A 801–839 (*N* = 0.82 ± 0.03) (Fig. 1C). This ratio is consistent with the pull-down result that the trimeric assembly is not required for efficient binding. An alanine scan of the ATG9A tail (830–839) was performed by mutating single or double residues in the context of an MBP-tagged full-length ATG9 construct (Fig. 1C). Using an amylose pull-down assay, we determine the effect of tail residue side chains on ATG13:ATG101 binding. Recruitment of wild-type MBP-ATG9A, V839A, V836A, Q835A, L832A, and a double mutant (DE830-831AA) was robust in the presence of ATG13^HORMA^:ATG101 (Fig. 1, D and E). Mutants K838A and P833A reduced the binding efficiency to half when compared to wild type. The mutations H837A and P834A abolished the binding of ATG13^HORMA^:ATG101, reducing it to 20 and 5% of the wild type, respectively. Pull-down of an MBP-ATG9 (1–830), which lacks the tail completely, similarly abolished the interaction. These data identified the ATG9A tail (830–839) as the site of direct interaction between ATG13^HORMA^:ATG101, linking ATG9A to the ULK1 complex.

### Crystal structure of the ATG9A HDIR:ATG13^HORMA^:ATG101 ternary complex

**Fig. 2.**
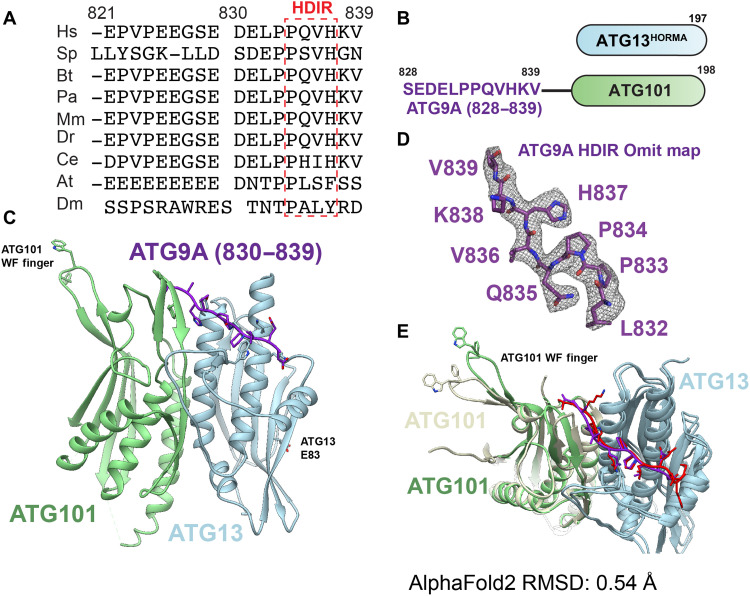
Ternary ATG9A HDIR:ATG13^HORMA^:ATG101 crystal structure. (**A**) Multisequence alignment of ATG9A C-terminal tails performed using ClustalO and aligned to ATG9A from Hs. Hs, *Homo sapiens*; Sp, *Schizosaccharomyces pombe*; Bt, *Bos taurus*; Pa, *Pongo abelii*; Mm, *Mus musculus*; Dr, *Danio rerio*; Ce, *Caenorhabditis elegans*; At, *Arabidopsis thaliana*; Dm, *Drosophila melanogaster*. (**B**) Constructs used for crystallization including ATG13^HORMA^ (1–197) and fused ATG9A (829–839)–GlySerLinker-ATG101 (1–198). (**C**) Ribbon representation of the ATG9A HORMA dimer–interacting region (HDIR)–ATG101 and ATG13^HORMA^ constructs for crystallography colored by protein. ATG13^HORMA^, light blue; ATG101, light green; ATG9A, purple. (**D**) Omit map of the ATG9A HDIR shown at 2.9σ. (**E**) Overlay of the crystal structure and AlphaFold2 (AF2)–predicted model aligned to the ATG13^HORMA^ domain. Crystal structure colored as in (C); AF2 model colored as follows: ATG13^HORMA^, light blue; ATG101, yellow; and ATG9A tail, red. RMSD, root mean square deviation.

Having mapped the HDIR, we pursued structural analysis of the ternary complex. Multisequence alignment showed that the residues within the HDIR are conserved across the Opisthokonta, with the exception being *Saccharomyces cerevisiae*, whose Atg9 ortholog has no sequence homology with the human HDIR (Fig. 2A). P834 and H837 are highly conserved across Opisthokonta ATG9A HDIR sequences, excluding budding yeasts. AlphaFold2 (AF2) predictive modeling (*53*) was performed using the primary sequence of the ATG13^HORMA^, ATG101, and ATG9A tail residues (828–839). To generate a set of models, the primary amino acid sequences of ATG9A, ATG13^HORMA^, and ATG101 were submitted in varying input positions. Thirty models were generated, which were overlaid to predict the position of the HDIR (fig. S1A). Two distinct positions were observed, one that involved binding only to ATG13^HORMA^ and one that spanned both HORMA domains. The models with the HDIR at the dimer interface had higher predicted local distance difference test scores. From these models, the HDIR was located adjacent to the N terminus of ATG101. We designed a fusion construct between the HDIR and ATG101, using a flexible linker of five residues (GSDEA) to increase the affinity of the ternary complex for crystallization (Fig. 2B).

We determined the crystal structure of the ATG9A HDIR-ATG101 fusion construct bound to ATG13^HORMA^ to 2.4-Å resolution (Fig. 2C and Table 1). Molecular replacement was performed using the ATG13^HORMA^:ATG101 apo structure [Protein Data Bank (PDB): 5C50] (*20*). The asymmetric unit contains two ATG9A HDIR:ATG13^HORMA^:ATG101 complexes, both in a similar conformation, except that the ATG101 WF finger is in different conformations in the two complexes due to differences in the crystal packing environment. ATG13^HORMA^ and ATG101 are in essentially the same conformation as in the apo ATG13^HORMA^:ATG101 structure with a root mean square deviation (RMSD) of 0.45 Å across all backbone atoms (fig. S1, B to E). Electron density at the interface for ATG13^HORMA^:ATG101 fit all 10 residues of the HDIR, allowing confident positioning of residues 830–839 (Fig. 2D). The ATG13^HORMA^:ATG9A and ATG101:ATG9A interfaces bury 388 and 241 Å^2^ of solvent-accessible surface area, respectively. Overall, our experimental structure agrees with the AF2 prediction with an RMSD of 0.54 Å for backbone atoms (Fig. 2E). Our ternary crystal structure of the ATG9A HDIR:ATG13^HORMA^:ATG101 complex determined that the HDIR binds at the interface between ATG13 and ATG101 HORMA domains.

**Table 1.** Data collection and refinement statistics. Values in parentheses are for the highest-resolution shell. ATG9, autophagy-related protein 9A; HDIR, Hop1/Rev7/Mad2 dimer–interacting region; RMS, root mean square.

	ATG9 HDIR:ATG101:ATG13 complex
**Data collection**	
Space group	*P*2_1_2_1_2_1_
Cell dimensions	
*a*, *b*, *c* (Å)	45.45, 139.86, 147.60
α, β, γ (°)	90, 90, 90
Resolution (Å)	39.41–2.41 (2.496–2.41)
*R*_pim_	0.03972 (0.5303)
*I*/σ*I*	14.55 (1.31)
*CC_1/2_*	0.999 (0.559)
Completeness (%)	99.75 (99.89)
Redundancy	6.6 (6.8)
	
**Refinement**	
Resolution (Å)	39.41–2.41
No. of reflections	37,256 (3659)
*R*_work_/*R*_free_ (%)	21.3 (29.4)/26.1 (35.6)
No. of atoms	6307
Protein	5867
Ligand/ion	252
Water	188
*B*-factors	64.5
Protein	63.44
Ligand/ion	89.94
Water	64.17
RMS deviations	
Bond lengths (Å)	0.005
Bond angles (°)	0.70
Clash score	5.56
Ramachandran plot	
Favored (%)	96.95
Allowed (%)	2.52
Outliers (%)	0.53

### ATG13^HORMA^ and ATG101 form a hydrophobic groove for ATG9A HDIR binding

**Fig. 3.**
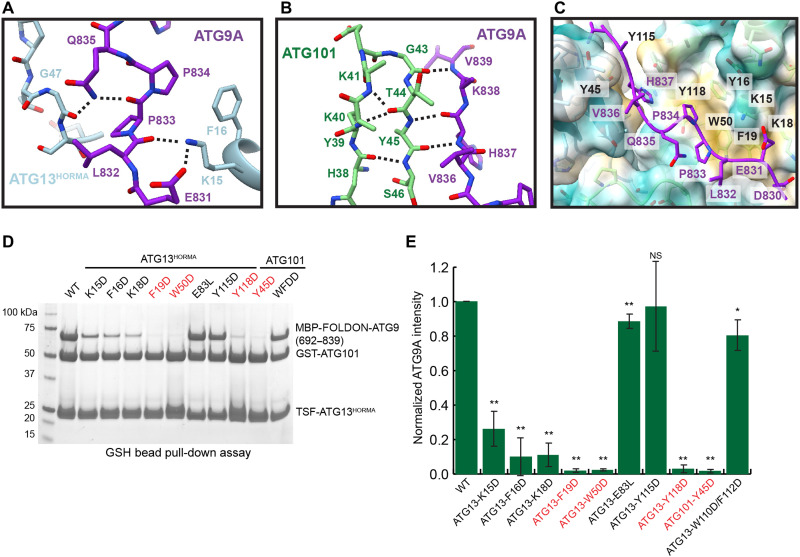
Interfaces between ATG9A HIDR and ATG13^HORMA^:ATG101. (**A**) Close-up of the hydrogen network between ATG13^HORMA^ and the ATG9A HDIR. (**B**) The extended β sheet between ATG101 and ATG9A. (**C**) View of the hydrophobic groove across the HORMA dimer interface, colored by hydrophobicity. (**D**) GST pull-down assay using purified wild-type TSF-ATG13^HORMA^:GST-ATG101 and listed mutants incubated with lysate containing MBP-FOLDON-ATG9A. Position of mutants E83 and WF finger shown in Fig. 2C. (**E**) Quantification of GST pull-down assays from (D) showing SD (*n* = 3). Two-sample *t* test was used to compare wild type to mutant pull-down results. *P* values are labeled as follows: *P < 0.05; **P < 0.005.

The interaction of the HDIR with the ATG13^HORMA^:ATG101 dimer is mediated by backbone and side-chain interactions. The loop between β2′ and αB in ATG13^HORMA^ (T46-W50) is positioned to support the binding of the HDIR (Fig. 3A). This rearrangement supports a hydrogen bonding interaction between ATG9A L832 and Q835 and ATG13 K15 and G47 (Fig. 3A). The loop region of G43-Y45 in ATG101 joins a β sheet network between β2 and HDIR residues H837-V839 (Fig. 3B). We termed this previously unidentified β sheet as β1′ (fig. S1). These two conformational changes in ATG13^HORMA^:ATG101 occur in the presence of ATG9A binding and are not observed in apo ATG13^HORMA^:ATG101 structures. The HDIR peptide binds across both HORMA domains within a long hydrophobic groove (Fig. 3C). ATG101 β2′ (Y45) and ATG13 αA (K15, F16, K18, and F19), αA-B connector (W50), and αC (Y115 and Y118) form a hydrophobic patch of residues, which support the full length of the HDIR.

To validate the ATG13^HORMA^:ATG101:HDIR interface, we used a glutathione *S*-transferase (GST) pull-down assay to monitor the recruitment of ATG9A (692–839) to wild- type and mutated ATG13^HORMA^:ATG101 proteins. Wild-type ATG13^HORMA^:ATG101 robustly recruited ATG9A (Fig. 3, D and E). Mutations of hydrophobic residues in ATG13^HORMA^ (K15D, F16D, and K18D) impaired ATG9A binding. Removing the hydrophobic residues, F19D, W50D, and Y118D in ATG13^HORMA^ and Y45D in ATG101 fully abolished the binding of ATG9A. Mutations of Y115D and E83L in ATG13^HORMA^, which is homologous to the putative binding site of budding yeast Atg13 to Atg9, had no effect on binding (*54*). Furthermore, removing the WF finger of ATG101 (WF to DD) had no effect on ATG9A binding. Our mutational analysis suggests that the hydrophobic groove drives ATG9A binding and explains the need for both ATG101 and ATG13 in binding ATG9A in vivo (*45*).

### ATG9A HDIR binding to ATG13^HORMA^:ATG101 facilitates NDP52-dependent mitophagy initiation

**Fig. 4.**
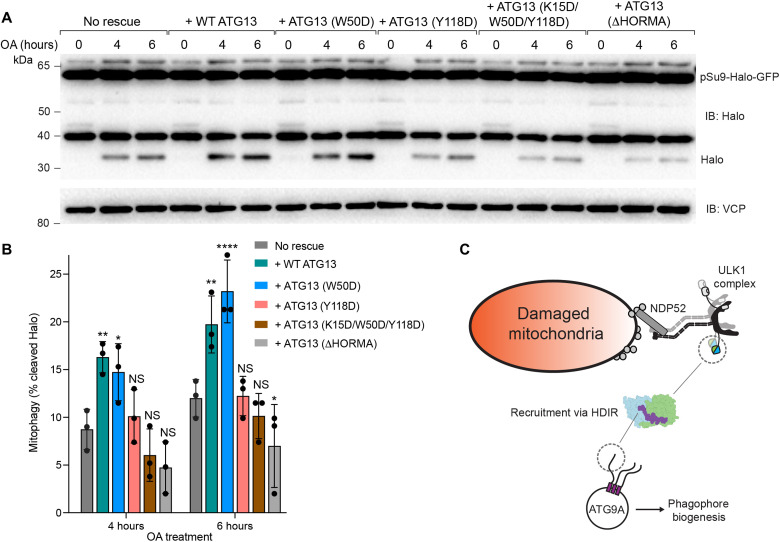
Pulse-chased pSu9-Halo-mGFP mitophagy reporter rescue experiments and model for ATG9:ATG13:ATG101-mediated mitophagy initiation. *ATG13* knockout (KO)/penta KO cells without rescue or rescued with different versions of HA-tagged ATG13 were incubated with 50 nM TMR-conjugated Halo ligand for 20 min. After this, cells were harvested [0 hours of oligomycin antimycin (OA) treatment] or treated with OA for indicated time periods and analyzed by immunoblotting (IB) (**A**), and the percentage of the cleaved Halo band was quantified (**B**). All cell lines are expressing BFP-Parkin, GFP-NDP52, and the mitochondrially targeted Halo construct (pSu9-Halo-GFP). Data in (B) are means ± SD from three independent experiments. *P < 0.05; **P < 0.005; ****P < 0.0001 [two-way analysis of variance (ANOVA)]. (**C**) Model schematic of ATG9:ATG13:ATG101 interaction in NDP52-dependent mitophagy.

We sought to determine whether the HDIR interaction with the HORMA dimer plays a role in PINK1/Parkin mitophagy initiation. We focused on mitophagy because of the evidence that the ULK1 complex (*13*, *15*) mediates ATG9A recruitment. The *ATG13* gene was deleted in the context of the mitophagy receptor penta-KO (OPTN, NDP52, TAX1BP1, p62, and NBR1) cell line (fig. S2A) (*48*). The *ATG13* gene was deleted in the context of the inner mitochondrial matrix reporter pSu9-Halo-mGFP cell line, which measures Halo^R^ as a reporter of mitophagy flux (*55*). In the absence of ATG13, mitochondrial degradation was impaired as shown by the lack of free Halo^R^ at 4 and 6 hours of oligomycin-antimycin treatment (Fig. 4, A and B). Rescue experiments with hemagglutinin tagged wild-type ATG13 (HA_-ATG13) showed a recovery of mitophagy flux to reported levels (*55*). Rescue experiments using ATG13 Y118D mutant, triple mutant (K15D, W50D, and Y118D), and ΔHORMA ATG13 did not recover the loss of mitophagy flux (Fig. 4, A and B). Expression of ATG13 W50D did rescue NDP52-dependent mitophagy, however (Fig. 4, A and B), which could be due to a difference in the stringency of the in vitro binding and in cellulo mitophagy assay. Expression levels of ATG13 wild type and mutants were comparable throughout all experiments (fig. S2A). These findings demonstrate that the HDIR binding interface on ATG13 contributes to PINK1/Parkin mitophagy.

## DISCUSSION

Understanding the interactions responsible for autophagy initiation is central to efforts to therapeutically modulate bulk and selective autophagy pathways and to understand their functioning at a fundamental level. Here, we determined the structure of the ATG9A HDIR bound to the ATG13:ATG101 HORMA dimer (Fig. 2C). As a central part of the autophagy initiating the ULK1 complex, the HORMA dimer is considered a key element, enabling the ULK1 complex to recruit ATG9A vesicles for autophagy initiation (*18*, *19*, *44*). The structure helps bring an understanding of ATG9A binding in the context of other structural information on the HORMA dimer and HORMA domains in general. The HDIR site overlaps a hydrophobic binding site for the small-molecule benzamidine that was identified in the apo structure of the human HORMA dimer (*20*), explaining the normal function of this site. Other HORMA domain proteins function as protein interaction hubs regulated by conformational changes in the safety belt region (*22*). This posed the question whether interactions of the ATG13:ATG101 dimer could be regulated by conformational switching as seen for Mad2 and other HORMA domains (*22*). The interaction between the ATG9A HDIR and ATG13:ATG101 is distal to and thus apparently independent of the safety belt region.

Our biochemical experiments confirmed that the ATG13 residues that bind the ATG9A HDIR in the structure are required for full function. We noticed that none of these mutations completely abolishes mitophagy (Fig. 4, A and B). On the other hand, the deletion of the entire ATG13 HORMA domain has a nearly complete loss of function, as seen by Kannangara *et al*. (*45*). In our Halo assay, mutation of ATG13 W50D, which disrupted the HDIR interaction in vitro (Fig. 3D), had no in cellulo defect (Fig. 4, A and B). This discrepancy may arise from technical differences in the assays or it may point toward an unknown function of W50D. Either way, further work is required to fully elucidate the function of this site on ATG13. In addition, the HORMA dimer has additional functions distinct from the ATG9A tail binding pocket, which may play a role. We confirmed that the essential WF motif of ATG101 (*21*) is not involved in ATG9A binding. An intact ATG13 HORMA dimer is required for ATG101 to associate with the rest of the ULK1 complex via the ATG13 IDR (*20*). The unaccounted-for residual function of the HORMA dimer seems likely related to the role of the WF finger, which will be important to establish.

Human ATG9A contains a long C-terminal IDR, whereas budding yeast Atg9 contains an extensive N-terminal IDR. Sequence alignment of the ATG9A tail shows a strong conservation across most of the Opisthokonta (Fig. 2A) except budding yeast, which lacks the HDIR. In addition to ATG9A, mammals express the homologous ATG9B in a few tissues; however, ATG9B does not contain the HDIR motif. The physiology of human ATG9B is relatively little explored; thus, the importance of this difference is currently hard to interpret. The mammalian *S. cerevisiae*, as well as related thermophilic yeasts such as *Kluyveromyces lactis* and *Lachancea thermotolerans* that have been used as model systems in autophagy, has distinct initiation machinery when compared to other Opisthokonta (*7*). The ULK1 (human) kinase complexes contain ATG13 along with its constituent binding partner ATG101. However, budding yeast entirely lacks an ATG101 ortholog, and its functions are apparently replaced by the single HORMA domain in yeast Atg13 (*56*). Thus, although budding yeast Atg9 binds to the HORMA domain of Atg13 (*54*), the mammalian sequence motif and structural interactions do not seem to exist in yeast.

We combined biochemical analysis and predictive modeling to generate a fusion ATG9A-ATG101 protein for experimental structure determination. Protein structure prediction has rapidly advanced via deep learning algorithms such as AF2 (*53*, *57*). Predictive modeling can determine the binding pockets of polypeptide chains with an RMSD of <2 Å (*58*). However, validation of structural models remains a critical step in guiding our understanding of biological mechanisms. Minimizing the amount of primary sequence during modeling can greatly improve the accuracy of predicted interfaces. Our truncation data limited the primary sequence for the ATG9A component to be modeled on the ATG13:ATG101 dimer, making AF2 prediction of the complex feasible. Determining which of the two modeled binding sites was correct relied on experimental information. The potential for predictive models combined with in vitro biochemical analysis to correctly determine protein-protein interfaces will continue to grow as more methods are explored and structural studies are guided by predictive models. In our study, we found that experimental interaction mapping provided essential constraints before AF2 prediction of the complex, and downstream model validation by mutational and structural analysis was important to establish confidence in the model.

Ubiquitinated autophagy cargoes recruit receptors (OPTN, p62, NBR1, NDP52, and TAX1BP1) and some bind directly to the ULK1 complex via FIP200 (*13*–*15*). This includes NDP52 recognition of Parkin ubiquitination marks on mitochondria damaged by uncoupling agents (*13*). These interactions drive clustering and allosteric activation (*16*) of the ULK1 complex to promote the autophagy initiation cascade. While the micromolar affinity of the ATG9A HDIR:ATG13:ATG101 interaction is moderate, it is easy to see how the clustering of multiple ULK1 complexes on an extended ubiquitinated platform such as a damaged mitochondrion would contribute avidity to recruitment of ATG9A trimers. The 1:1 stoichiometry established here implies that a single ATG9A trimer can bind to three ULK1 complexes presented in a cluster on a cargo substrate (Fig. 4C). The interaction between the C-terminal IDRs of the ATG13 molecules and the N-terminal crescent domains of the FIP200 molecules would position the massive C-terminal coiled coil domains of FIP200 distal to the ATG9A vesicles. On the basis of the dimensions of FIP200 (*24*), this would allow for fly casting for ATG9A vesicles as far as roughly 35 nm from sites of clustered ubiquitin.

While our model presents a satisfying and plausible model for ATG9A vesicle recruitment downstream of the ULK1 complex in autophagy initiation, this is almost certainly not the complete mechanism or the only mechanism. Further mechanistic research is required to elucidate the role of the ATG9A HDIR motif on both selective and bulk autophagy pathways outside of mitophagy. Our attempts to perform KO and rescue experiments of ATG9A failed due to known mislocalization of ATG9A upon overexpression. It remains to be determined whether the ATG9A HDIR is the sole interactor at the ATG13-ATG101 interface. Kannangara *et al*. (*45*) found that ULK1 could still interact with ATG9A in the absence of ATG13 as measured by BioID, although this was insufficient to support normal levels of p62 autophagy. Thus, at least one additional point of contact between ATG9A and the ULK1 complex is likely to exist, at least in some forms of autophagy. A more significant departure from this mechanism is suggested by the finding that OPTN can recruit ATG9A directly, bypassing the need for the ATG9A-binding role of the ULK1 complex (*50*). The physiological rationale for these various pathways remains largely unknown, but the distinct role of different cargo receptors in neurodegeneration suggests that it will be important to better understand. Elucidating the molecular details of distinct initiation mechanisms in various “flavors” of mitophagy should provide windows into disease mechanism and ultimately therapy.

## MATERIALS AND METHODS

### Plasmid construction

Plasmids were engineered and modified according to the published protocol (dx.doi.org/10.17504/protocols.io.bxrjpm4n). The fusion construct of human ATG9A C terminus with ATG101 for crystallization was subcloned as an N-terminal GST tag, Tobacco etch virus (TEV) cleavage site, ATG9 (828–839), and 5–amino acid linker (GSDEA), followed by ATG101 (1–198) into the pCAG-eGFP vector [Research Resource Identifier (RRID): Addgene_89684]. ATG13 (1–197) (no tag) was subcloned into the pCAG vector using Cla I/Xho I sites. The ATG101:ATG13 HORMA construct for the ITC experiment was previously described (*20*). Proteins were tagged with GST, MBP, or TwinStrep-Flag (TSF) for affinity purification or pull-down assays. All constructs were verified by DNA sequencing. Plasmids are deposited to Addgene.org [pCAG-GST-ATG9 (828-839)-ATG101 (1-198) (RRID:Addgene_188073); pCAG-ATG13 (1-197) (RRID:Addgene_188074); pCAG-OSF-ATG13 (2-197) (RRID:Addgene_188075); pCAG-MBP-ATG9 (RRID:Addgene_188076); pCAG-MBP-ATG9-V839A (RRID:Addgene_188077); pCAG-MBP-ATG9-K838A (RRID:Addgene_188078); pCAG-MBP-ATG9-H837A (RRID:Addgene_188079); pCAG-MBP-ATG9-V836A (RRID:Addgene_188080); pCAG-MBP-ATG9-Q835A (RRID:Addgene_188081); pCAG-MBP-ATG9-P834A (RRID:Addgene_188082); pCAG-MBP-ATG9-P833A (RRID:Addgene_188083); pCAG-MBP-ATG9-L832A (RRID:Addgene_188084); pCAG-MBP-ATG9-D830A/E831A (RRID:Addgene_188085); pCAG-MBP-ATG9 (1-830) (RRID:Addgene_188086); pCAG-MBP-Foldon-ATG9 (692-839) (RRID:Addgene_188087); pCAG-MBP-Foldon-ATG9 (723-839) (RRID:Addgene_188088); pCAG-MBP-Foldon-ATG9 (801-839) (RRID:Addgene_188089); pCAG-MBP-Foldon-ATG9 (828-839) (RRID:Addgene_188090); pCAG-MBP-Foldon-ATG9 (830-839) (RRID:Addgene_188091); pCAG-MBP-ATG9 (692-839) (RRID:Addgene_188092); pCAG-MBP-ATG9 (801-839) (RRID:Addgene_188093); pCAG-OSF-ATG13 (2-197)-K15D (RRID:Addgene_188094); pCAG-OSF-ATG13 (2-197)-F16D (RRID:Addgene_188095); pCAG-OSF-ATG13 (2-197)-K18D (RRID:Addgene_188096); pCAG-OSF-ATG13 (2-197)-F19D (RRID:Addgene_188097); pCAG-OSF-ATG13 (2-197)-W50D (RRID:Addgene_188098); pCAG-OSF-ATG13 (2-197)-E83L (RRID:Addgene_188099); pCAG-OSF-ATG13 (2-197)-Y115D (RRID:Addgene_188100); pCAG-OSF-ATG13 (2-197)-Y118D (RRID:Addgene_188101); pCAG-OSF-ATG13 (2-197)-L151D (RRID:Addgene_188102); pCAG-OSF-ATG13 (2-197)-Y152D (RRID:Addgene_188103); pCAG-OSF-ATG13 (2-197)-R153D (RRID:Addgene_188104); pCAG-GST-ATG101-Y45D (RRID:Addgene_188105); pCAG-GST-ATG101-W110D/F112D (RRID:Addgene_188106)].

### Protein expression and purification

For crystallization, GST-ATG9 (828–839)–ATG101 were cotransfected with ATG13 (1–197) using the polyethylenimine-MAX (Polysciences) transfection system. Cells were transfected at a concentration of 2 × 10^6^ ml^−1^. After 48 hours, cells were pelleted at 500*g* for 10 min, washed with phosphate-buffered saline (PBS) once, and then stored at −80°C. The pellets were lysed with lysis buffer [25 mM Hepes (pH 7.5), 200 mM NaCl, 2 mM MgCl_2_, 1 mM tris(2-carboxyethyl)phosphine (TCEP), 5 mM EDTA, and 10% glycerol] with 1% Triton X-100 and protease inhibitor cocktail (Thermo Fisher Scientific, Waltham, MA) before being cleared at 17,000 rpm for 35 min at 4°C. The clarified supernatant was purified on GST Sepharose 4B resin (GE Healthcare) and then eluted in the lysis buffer with 25 mM glutathione. A general protocol for purification of GST-tagged ATG13:ATG101 constructs has been uploaded online (dx.doi.org/10.17504/protocols.io.cc53sy8n). After His_6_-TEV cleavage at 4°C overnight, the sample was diluted five times in HiTrap SP HP column buffer A [30 mM MES (pH 6.0) and 3 mM β-mercaptoethanol (BME)] and then loaded onto a HiTrap SP HP 5-ml column (GE Healthcare, Piscataway, NJ). Elution from the SP column was performed with a 70-ml linear gradient from 0 to 500 mM NaCl in SP buffer A. The cleavage sample was eluted at the buffer conductivity of ~25 mS/cm. After each fraction was analyzed by SDS gel, the pooled fractions were concentrated in an Amicon Ultra-15 concentrator (MilliporeSigma, Burlington, MA) and exchanged buffer with the ITC buffer [25 mM Hepes (pH 7.5), 150 mM NaCl, 1 mM MgCl_2_, and 1 mM TCEP].

For the ITC experiment, the GST-ATG13 (12–200):ATG101 (1–198) complex was expressed in *Spodoptera frugiperda* (Sf9) cells [American Type Culture Collection (ATCC) CRL-1711; RRID: CVCL_0549]. Baculoviruses were generated in Sf9 cells with the Bac-to-Bac System (Life Technologies). Cells were infected and harvested after 72 hours. Purification was performed as described above for HEK cell expression.

MBP-ATG9A–related constructs were transfected in HEK GnTi^−^ cells at 2 × 10^6^ ml^−1^ density (ATCC CRL-3022; RRID: CVCL_A785). Cells were harvested after 48 hours of transfection. For ATG9A CTD constructs, the pellets were lysed with lysis buffer/protease inhibitor cocktail/1% Triton X-100 at room temperature for 15 min. The clarified supernatant was purified on amylose resin (New England Biolabs, Ipswich, MA) and then eluted in the lysis buffer with 40 mM maltose (Sigma-Aldrich, St. Louis, MA). The eluted samples were further concentrated and then loaded onto a Superose 6 Increase 10/300 GL column (Cytiva, Marlborough, MA) in ITC buffer. For MBP-ATG9A full protein, cells were lysed in lysis buffer/protease inhibitor cocktail/1% n-Dodecyl β-D-Maltopyranoside/Cholesterol Hydrogen Succinate (DDM/CHS). All wash, elution, or pull-down buffers contain 0.05% DDM/CHS. Purification of ATG9A truncations via amylose resin has been deposited online (dx.doi.org/10.17504/protocols.io.6qpvr6nkovmk/v1).

The TSF-ATG101:ATG13 (1–197) complex used for pull-down assay was expressed in HEK GnTi^−^ cells at 2 × 10^6^ ml^−1^ density. After 48 hours of transfection, the pellets were lysed with the lysis buffer/1% Triton X-100 at room temperature for 15 min. The clarified supernatant was purified on Strep-Tactin resin (IBA Lifesciences, Germany). After extensive wash, the sample was eluted with lysis buffer/4 mM desthiobiotin and then loaded onto a Superdex 200 Increase 10/300 GL column (Cytiva) in ITC buffer. The method for purification of TSF-tagged ATG13:ATG101 dimers has been uploaded online (dx.doi.org/10.17504/protocols.io.cc54sy8w).

### Bead pull-down assays

GST-ATG101:TSF-ATG13 (1–197) HORMA wild type or mutants were expressed in 10 ml of HEK GnTi cells. The cells were harvested 48 hours after transfection. The pellets were homogenized in 0.5 ml of lysis buffer/protease inhibitors/1% Triton X-100 and clarified after 40,000*g* for 15 min. The lysate was incubated with 30 μl of Glutathione-Sepharose beads (GE Healthcare) at 4°C for 4 hours. The beads were washed twice by lysis buffer and then incubated with recombinant MBP proteins (final concentration of 2 μM) or lysate from 10 ml of HEK cells at 4°C overnight. The next day, the beads were washed four times and then eluted in 50 μl of ITC buffer/25 mM glutathione. Seventeen microliters of eluent was mixed with lithium dodecyl sulfate (LDS)/BME buffer, heated at 60°C for 5 min, and subjected to SDS–polyacrylamide gel electrophoresis (SDS-PAGE) gel. The protocol for GST resin-based pull-down assay is deposited online (dx.doi.org/10.17504/protocols.io.kqdg3pdrpl25/v1).

In 300 μl, recombinant MBP proteins (final 1.2 μM) and the TSF-ATG101:ATG13 (1–197) complex (final 3 μM) were incubated with 30 μl of amylose resin (New England Biolabs, Ipswich, MA) at 4°C overnight in the ITC buffer. The beads were washed four times and then eluted in 50 μl of ITC buffer/50 mM maltose. Seventeen microliters of eluent was mixed with LDS/BME buffer, heated at 60°C for 5 min, and subjected to SDS-PAGE gel. Pull-downs were performed in three biological replicates, and band intensities were quantified using Fiji, version 2.3.0/1.53f (http://fiji.sc; RRID: SCR_002285). SD was calculated for the three pull-down assays and shown in bar graph form. Raw data from this assay have been uploaded to Zenodo (DOI: 10.5281/zenodo.7632198), and protocol has been published online (dx.doi.org/10.17504/protocols.io.ccw6sxhe). Statistical significance for bead-based assays was determined from using a two-sample *t* test. *P* < 0.05; error bars are means ± SD.

### Isothermal titration calorimetry

All samples were dialyzed against the ITC buffer at 4°C overnight. The sample cell contained 0.2 ml of 17 μM ATG101-ATG13 HORMA complex, and MBP-ATG9 (801–839) (240 μM) was added in 13 injections of 2.7 μl each. Measurements were repeated four times and carried out on a MicroCal PEAQ-ITC instrument according to the manual (Malvern Panalytical Inc., Westborough, MA) https://jeltsch.org/sites/jeltsch.org/files/files/MicroCal-PEAQ-ITC-user-manual-English-MAN0573-01-EN-00.pdf. The data were processed using MicroCal PEAQ-ITC 1.4.1 analysis software (www.malvernpanalytical.com/en/support/product-support/microcal-range/microcal-itc-range/microcal-itc200; RRID: SCR_020260). The binding constant (*K*_d_) was fitted using a one-site model.

### Modeling, crystallization, and crystallographic analysis

AlphaFold2 (https://alphafold.ebi.ac.uk/) was run using the ColabFold notebook (https://github.com/sokrypton/ColabFold) using version 2.1 on default settings. Multisequence alignment of Fig. 2A was performed in SeaView version 5.0.4 (www.mybiosoftware.com/seaview-4-2-12-sequence-alignment-phylogenetic-tree-building.html; RRID: SCR_015059) (*59*). The ATG9 HDIR (828–839)–fused ATG101 (1–198):ATG13 (1–197) complex was concentrated to 6 mg/ml in the ITC buffer. Crystallization was carried out by sitting drop vapor diffusion using an automated liquid handling system (Mosquito, TTP Labtech, UK) at 288 K in 96-well plates. The protein solution was mixed with the reservoir buffer composed of 0.1 M Hepes (pH 7.5), 0.2 M NaCl, and 12% PEG8000 (polyethylene glycol, molecular weight 800) with a ratio of 1:1. The crystal was obtained in 2 to 4 days. Crystals were cryoprotected in 28% glycerol/reservoir buffer and frozen in liquid N_2_. The protocol for purification of the ATG9 HDIR-ATG101:ATG13^HORMA^ complex and crystallization has been deposited (dx.doi.org/10.17504/protocols.io.cc55sy86).

Native data were collected from a single frozen crystal using a Dectris Pilatus 6M detector at beamline 12-2, Stanford Synchrotron Radiation Lightsource (SSRL). All data were processed and scaled using X-ray Detector Software 4.0 (https://xds.mr.mpg.de/; RRID: SCR_015652) (*60*). The crystal diffracted to 2.4-Å resolution and belonged to space group *P*2_1_2_1_2_1_ with unit cell dimensions *a* = 45.453 Å, *b* = 139.86 Å, *c* = 147.595 Å, and α = β = γ = 90°. A molecular replacement solution was found using partial structures derived from the ATG101:ATG13 HORMA apo structure (PDB: 5C50) as a search model with Phenix (version 1.20.1-4487) (*61*). Model building and refinement were carried out using Coot 0.9.6 EL (www2.mrc-lmb.cam.ac.uk/personal/pemsley/coot/; RRID: SCR_014222) (*62*) and Phenix version 1.20.1-4487 (www.phenix-online.org/; RRID: SCR_0142294) (*61*). Structural figures were generated with PyMOL, version 2.5 (https://pymol.org/; RRID: SCR_000305) (*63*) or UCSF Chimera, version 1.16 (http://plato.cgl.ucsf.edu/chimera/; RRID: SCR_004097) (*64*).

### Halo assay to assess mitophagy

Halo assay to measure mitophagy was recently described (*55*). We used the published pSu9-Halo-mGFP plasmid (Addgene, no. 184905) for our mitophagy assays. The detailed protocol is available online (dx.doi.org/10.17504/protocols.io.dm6gpjzm8gzp/v1). Mitophagy treatment was previously described (*65*). Briefly, penta KO.ATG13 KO cells (RRID: CVCL_C2VP) were seeded the day before the treatment day in six-well plates. The cells were then incubated with growth media [Dulbecco’s modified Eagle’s medium with 10% fetal bovine serum, glucose (4.5 g/liter; Sigma-Aldrich, G8769), 1× GlutaMAX (Thermo Fisher Scientific, 35050061), 1× MEM Non-Essential Amino Acid (Thermo Fisher Scientific, 11140-050), and 25 mM Hepes (1688449)] containing 50 nM tetramethylrhodamine (TMR)-conjugated Halo ligand for 20 min. The cells were then washed twice with 1× PBS. Untreated samples were harvested, while mitophagy-induced samples were treated with growth media containing 4 μM antimycin A (Sigma-Aldrich, A8674), 10 μM oligomycin (Calbiochem, 495455), and 10 μM Quinoline-Val-Asp-Difluorophenoxymethylketone (QVD) (MedChem Express, HY-12305) for the indicated time periods. Following treatment, the cells were washed with ice-cold 1× PBS, harvested using cell scrapers, and lysed in lysis buffer containing 1× LDS sample buffer (Life Technologies) and freshly added 100 mM dithiothreitol (Sigma-Aldrich). Samples were heated at 99°C with shaking for 7 min. Approximately 25 μg of protein per sample was run on 4 to 12% Bis-Tris gels (Life Technologies) according to the manufacturer’s instructions. Gels were electrotransferred to polyvinyl difluoride membranes and immunoblotted with antibodies against valosin-containing protein (VCP) (Cell Signaling Technology, Cat#2649; RRID: AB_2214629) and HALO (Promega, Cat#G9211; RRID: AB_2688011). For Western blot quantification, band intensities were measured with Image Lab 5.2.1 (RRID:SCR_014210, Bio-Rad, http://www.bio-rad.com/en-us/sku/1709690-image-lab-software). Statistical significance was calculated from three independent experiments using two-way analysis of variance (ANOVA). Error bars are means ± SD.

## Notes

View/request a protocol for this paper from *Bio-protocol*.
