# Supplementary material for: Structural basis for ATG9A recruitment to the ULK1 complex in mitophagy initiation
Source: Sci Adv. 2023 Feb 15;9(7):eadg2997. doi: 10.1126/sciadv.adg2997 (PMC9931213; doi:10.1126/sciadv.adg2997)
Supplement: Supplementary file 1 — Figs. S1 and S2 [file sciadv.adg2997_sm.pdf]

Science Advances
AAAS

Supplementary Materials for

Structural basis for ATG9A recruitment to the ULK1 complex in mitophagy initiation

Xuefeng Ren *et al.*

Corresponding author: James H. Hurley, jimhurley@berkeley.edu; Adam L. Yokom, ayokom@missouri.edu

Sci. Adv. **9**, eadg2997 (2023)
DOI: 10.1126/sciadv.adg2997

This PDF file includes:

Figs. S1 and S2

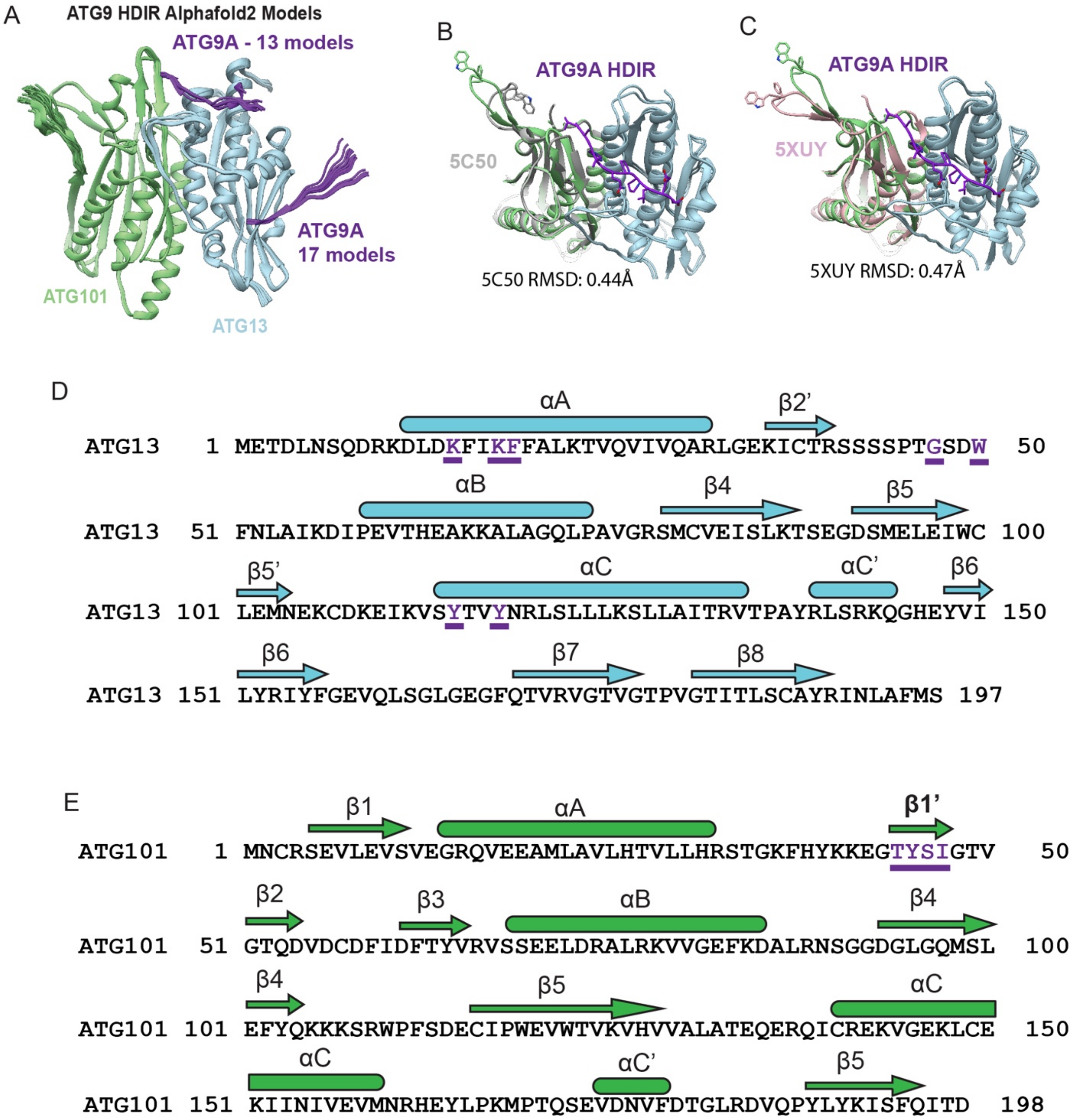

Figure S1 – Structural data for ATG9 HDIR-ATG101:ATG13

A) Overlaid Alphafold2 models colors as in Figure 2 B) and C) Comparison of the ATG9 HDIR-ATG101:ATG13 to apo ATG101:ATG13 structures, PDB:5C50 and PDB:5XUY, respectively. D) Secondary structure of ATG13^{HORMA} with residues that interact with ATG9 HDIR shown in purple E) Secondary structure of ATG101 with residues that interact with ATG9 HDIR shown in purple

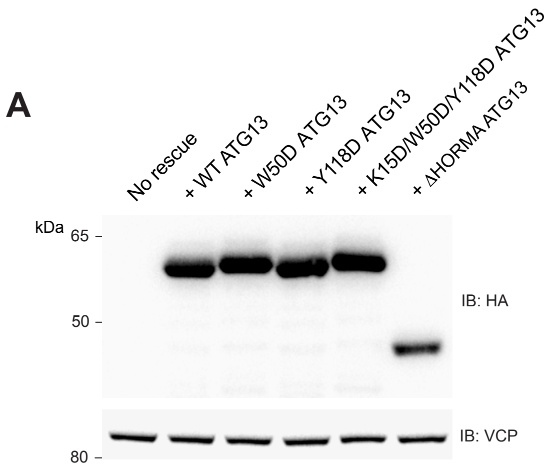

Figure S2 – Expression levels of ATG13 rescue in penta KO + NDP52 cell lines

A) Total cell lysates from indicated cell lines were immunoblotted with anti-HA and anti-VCP antibodies.
